# The role of vaccines in the COVID-19 pandemic: what have we learned?

**DOI:** 10.1007/s00281-023-00996-2

**Published:** 2023-07-12

**Authors:** Florian Krammer

**Affiliations:** 1 Department of Microbiology, Icahn School of Medicine at Mount Sinai, New York, NY USA; 2 Center for Vaccine Research and Pandemic Preparedness (C-VaRPP), Icahn School of Medicine at Mount Sinai, New York, NY USA; 3 Department of Pathology, Molecular and Cell Based Medicine, Icahn School of Medicine at Mount Sinai, New York, NY USA

**Keywords:** SARS-CoV-2, Vaccines, COVID-19, Pandemic, Coronavirus

## Abstract

Severe acute respiratory syndrome coronavirus 2 (SARS-CoV-2) emerged late in 2019 and caused the coronavirus disease 2019 (COVID-19) pandemic that has so far claimed approximately 20 million lives. Vaccines were developed quickly, became available in the end of 2020, and had a tremendous impact on protection from SARS-CoV-2 mortality but with emerging variants the impact on morbidity was diminished. Here I review what we learned from COVID-19 from a vaccinologist’s perspective.

## Introduction

Late in December of 2019 Chinese authorities reported an outbreak of pneumonia with unknown etiology in Wuhan, Hubei province, to the World Health Organization [1]. A few days later, on January 10, 2020 (at 8:07 pm Eastern Standard Time to be exact [personal communication by Edward Holmes]; January 11th Australian Eastern Standard Time), the sequence of the causative agent was posted online by Edward Holmes (University of Sydney) on behalf of a consortium led by professor Yong-Zhen Zhang (Fudan University) [2]. It turned out to be a coronavirus, provisionally named nCoV (new coronavirus) and later named severe acute respiratory syndrome coronavirus 2 (SARS-CoV-2) due to its similarities with SARS-CoV-1. Case numbers locally increased quickly [3], and the virus was already causing community transmission in several WHO regions [4–7] before the WHO finally declared a pandemic on March 11, 2020. The outbreak did not come without warning. In 2002/2003, SARS-CoV-1 caused an outbreak which went global but could be stopped at approximately 8000 cases using non-pharmaceutical interventions [8]. In 2012, the Middle Eastern respiratory syndrome (MERS) coronavirus was detected for the first time in Saudi Arabia [9], and a large MERS-CoV outbreak in 2015 in South Korea was stopped, again with rigorous non-pharmaceutical interventions. In addition, OC43, a human seasonal coronavirus closely related to bovine coronavirus, is hypothesized to have caused the “Russian flu” pandemic of 1889/1890 [10]. In addition, surveillance in wildlife in Southeast Asia had shown that SARS-like coronaviruses that readily infected human cells were circulating in bats in the area [11] and serosurveillance studies [12] in humans made it clear that there was large-scale exposure to these viruses. One paper from 2016 literally had the title “A SARS-like cluster of circulating bat coronaviruses shows potential for human emergence” [11]. It is therefore no surprise that vaccinologists had been developing vaccine strategies for these viruses. Two vaccines for SARS-CoV-1 made it to human clinical trials, including a DNA vaccine developed in the USA and an inactivated whole-virus vaccine developed in China [13]. However, these vaccines were not advanced any further since SARS-CoV-1 had been eliminated from the human population and no further zoonotic spillovers occurred. There were also concerns about vaccine enhanced disease which had been observed in some animal models for both SARS-CoV-1 and MERS-CoV [14]. Nevertheless, pre-clinical vaccine development and antigen design for coronaviruses continued and provided important insights later on for SARS-CoV-2 vaccine development [15, 16].

## SARS-CoV-2 vaccine development and implementation

With the discovery of the causative agent and the sequencing of its genome, the race towards a SARS-CoV-2 vaccine started in early January 2020. A large number of entities initiated vaccine development in parallel using a wide variety of vaccine platforms [14]. The focus of many of these vaccines was the spike protein of SARS-CoV-2 which engages with the angiotensin converting enzyme 2 (ACE2) on host cells [17]. The rational for many vaccines was that antibodies to the spike could block this interaction, thereby neutralizing the virus and providing protection. In March 2020, the first phase I clinical trials started rapidly (Table 1) followed by phase II and phase III trials with first phase III trial results available in November 2020. However, several vaccines were already licensed based on phase II studies for limited use in the population including the replication incompetent adenovirus 26/adenovirus 5 vector combination “Sputnik” in Russia and CanSino’s adenovirus 5 vectored vaccine in China [18, 19]. Large-scale rollout of vaccines started in December of 2020 with the mRNA vaccine of Pfizer/BioNTech followed by Moderna’s mRNA vaccine and AstraZeneca’s vectored vaccine (Table 1). Times from initial phase I trials to first approval varied from 2 months (Sputnik V, viral vector) to 19 months (Novavax, recombinant spike). These wide intervals were influenced by differences in the rigor of regulatory systems, clinical trial design, and vaccine platform. In general, vectored, mRNA, and inactivated vaccines as well as recombinant receptor-binding domain (RBD) vaccines tended to be faster than recombinant spike and virus-like particle (VLP) vaccines (Table 1). One of the strongest influences for speed of initial licensure of the “ultra-fast” vaccines (licensed in less than 6 months) seems how “flexible” regulatory systems in the respective countries are and how little safety data was necessary for initial licensure. For some vaccines, e.g., Sputnik V, this leaves a big question mark. Initial reported vaccine efficacies were high for most of the vaccine candidates, in many cases above 90% against symptomatic SARS-CoV-2 infections (Table 1). Of note, vaccine efficacy is assessed in phase III clinical trials by calculating the percentage of reduction of disease in the vaccine group versus the control group. Importantly, endpoints of clinical trials for different vaccines can differ, which may complicate cross-comparison between vaccines. Initially, protection against asymptomatic infection was also reported [20], at least for one mRNA vaccine candidate. However, emergence of viral variants in late 2020 led to lower vaccine efficacy for several of the candidates that entered phase III trials late but also gave an opportunity to determine vaccine efficacy against new variants. In addition, initial vaccine candidates were designed either as two-dose or even one dose regimens but the emergence of variants and waning immunity made booster doses necessary (e.g., [21]). After the initial rollout, in late 2020 and early 2021, the Delta variant emerged in India causing a global wave of infections. During this time, first breakthrough infections and transmission chains between vaccinated individuals were detected at somewhat larger scale [22]. As mentioned above, the lower efficacy of the vaccines during that time may have had several reasons. One is the somewhat waning immunity, and with that less of the initially measurable (neutralizing) IgG antibody on mucosal surfaces of the upper respiratory tract which can eliminate incoming virus [23] (see the Mucosal immunity section for a more in-depth discussion of this topic). However, this may not have been the main reason. The Delta variant showed some degree of immune escape due to mutations in the RBD and N-terminal domain (NTD), but it had also a number of other features that made it more prone to cause breakthrough infections. It was causing higher virus loads in the upper respiratory tract meaning that infected people would shed more virus and their contacts would be exposed to higher infectious doses [24, 25]. It also was more fusogenic, perhaps even fusing at the cell membrane which may allow it to enter cells quicker and to escape mucosal antibody [26]. Finally, it also had a shorter incubation time meaning there was less time for an anamnestic response to kick in and remove the virus before symptom onset [27]. All these factors together likely explain the observed breakthroughs and onward transmission of Delta in vaccinated individuals. However, it has to be kept in mind that these events were still relatively rare. When Omicron emerged in November of 2021 and replaced the Delta variant, breakthrough infections and onward transmission became common [28], although a residual protective effect remained, especially in individuals with hybrid immunity [29, 30] (hybrid immunity refers to individuals who had both infections and vaccinations [31]). Omicron variants acquired extensive mutations in RBD and NTD which allow efficient escape from neutralizing antibodies and these viruses have an even shorter incubation period, both contributing to increased breakthrough infections [27, 32]. Of note, binding antibodies and T-cells induced by vaccination still recognize Omicron variants well [32] and do protect relatively efficiently from severe disease and death [33–35]. However, in order to again maximize protection, updated vaccines containing the BA.5 spike (plus the wild-type spike) have been licensed (see below).

**Table 1:** Overview of select vaccine candidates and when they were approved

Vaccine (company)	Clinical trials start	First approval^1^	Time from phase I to first approval	Country^2^	Type of vaccine	Spike modifications	Phase III efficacy	References
AD5-nCOV, Convidecia (CanSino)	March 16, 2020 NCT04313127	June 2020	3 months	China	Ad5 vectored	None	57.5%	[18, 135]
mRNA-1273, Spikevax (Moderna)	March 16, 2020 NCT04283461	December 2020	9 months	USA	mRNA	2P modification	94.1%	[51, 136]
Coronavac, PicoVac (Sinovac Biotech)	April 16, 2020 NCT04352608	August 2020 (for high-risk populations)	4 months	China	Inactivated whole-virus vaccine	None	50.7–83.5%	[137–140]
Covishield, Vaxzevria, AZD1222 (AstraZeneca)	April 23, 2020	December 2020	8 months	UK	ChAdOx vector	None	70.4	[49, 141]
Comirnaty, BNT162b2 (Pfizer/BioNTech)	April 23, 2020	December 2020	8 months	Germany	mRNA	2P modification	95%	[142]
BIBP COVID-19 vaccine, BBIBP-CorV (Sinopharm)	April 29, 2020	July 2020	3 months	China	Inactivated whole-virus vaccine	None	Approx. 74%	[143, 144]
NVX-CoV2373, Nuvoxid, Covovax (Novavax)	May 26, 2020 NCT04368988	December 2021	19 months	Australia/USA	Recombinant spike	2P modification, polybasic cleavage site removed	89.7%	[145, 146]
Sputnik V, Gam-COVID-Vac (Gamaleya Research Institute of Epidemiology and Microbiology)	June 18, 2020	August 2020	2 months	Russia	Ad5 vector followed by Ad26 vector	None	91.6%	[19, 147, 148]
CoVLP, Covifenz (Medicago)	July 10, 2020 NCT04450004	February 2022	18 months	Canada	Virus-like particle	2P modification, polybasic cleavage site removed	69.5%	[149, 150]
Covaxin, BBV152 (Bharat)	July 13, 2020 NCT04471519	January 2021	6 months	India	Inactivated whole-virus vaccine	None	77.8%	[151, 152]
Ad26.COV2.S, Jcovden (Janssen Vaccines/J&J)	July 15, 2020	February 2021	7 months	Belgium/USA	Ad26 vector	2P modification, polybasic cleavage site removed	66.9%	[153]
Soberana 02, FINLAY-FR-2 (Finlay Institute)	November 2, 2020	June 2021	7 months	Cuba	RBD conjugate	Not applicable	92%	[154, 155]
Corbevax (Biological E)	November 2020	December 2021	13 months	India	Recombinant RBD	Not applicable	Immuno-bridging	[156]
Abdala, CIGB-66 (Center for Genetic Engineering and Biotechnology)	December 7, 2020	July 2021	7 months	Cuba	Recombinant RBD	Not applicable	92.3%	[157, 158]
VLA2001 (Valneva)	December 16, 2020 NCT04671017	April 2022	16 months	UK	Inactivated whole-virus vaccine	None	Immuno-bridging, slightly superior to AZD1222	[159, 160]

^1^In some cases this is for high risk/special populations only (as indicated)

^2^Country were clinical development started

One issue during vaccine rollout was poor immune responses seen in individuals with certain underlying diseases who have a compromised immune system [36–39]. This is of course not unique for COVID-19 vaccines and has been observed for other vaccines as well. However, we need to understand better which conditions and treatments lead to reduced protection, which markers (e.g., antibody or T-cell responses) would indicate a suboptimal response and how we can then protect these individuals through alternative treatments (mAbs, anti-virals) or through non-pharmaceutical interventions, not just against SARS-CoV-2 but also future pandemic viruses.

The side-by-side comparison of many different vaccines during the pandemic that we have seen allows to draw some general conclusions about vaccine platforms. However, there are also a lot of caveats with these cross-comparisons as pointed out throughout the text. Of note, for mRNA-based vaccines, only vaccines with modified mRNA (N1-methylpseudouridine (m1Ψ)) have worked well while non-modified mRNA vaccines have not been licensed [40]. In terms of vaccine efficacy, it has become clear that, in general, mRNA and recombinant protein vaccines do best, followed by vectored vaccines (especially if given twice), followed by inactivate virus vaccines (Table 1; of note, while this is seen for SARS-CoV-2, it is not generalizable to other pathogens/vaccines). This is not only clear from efficacy studies but several large-scale effectiveness studies (meaning, how well vaccines work in the population after rollout compared to efficacy measured in clinical trials) confirm these trends as well [41–44]. This applies to protection from symptomatic disease. However, it has become clear as well that protection from severe disease is high with all applied vaccine platforms [42–44]. Interestingly, there are also differences in the dynamics of antibody responses after vaccination, with mRNA vaccines causing a strong peak post-vaccination with waning over several months and then a stabilization of titers, while for some of the adenovirus vaccines a slower but longer increase of antibody titers has been reported [45]. Another conclusion that can be drawn is about safety and reactogenicity. For reactogenicity, which is a term describing the mostly harmless short-term side effects triggered by innate immune responses [46], it was observed that both vectored vaccines and mRNA vaccines are on the upper range of what is usually seen for vaccines [47, 48]. Interestingly, reactogenicity for viral vectors seems to be higher after the first vaccination, while for mRNA it seems to be higher after the second immunization (suggesting a role for adaptive immunity) [49–51]. In many cases, vaccinees had to take sick days after the second shot of mRNA vaccines [52, 53]. This is not problematic for a pandemic response but mRNA vaccines likely need to be optimized to be less reactogenic for regular and more frequent administration. Another side effect detected, for which the mechanisms is unclear so far, are large delayed local reactions which seem to be harmless and occur mostly after the first dose of mRNA-1273 [54]. It has also become clear that the profile of rare but severe side effects differs between vaccine platforms. For adenoviral vectors, rare severe thrombotic events have been reported that occur mostly in younger women [55] while myocarditis in young men has been reported for mRNA vaccines [56] and rare anaphylactic reactions have been reported for both [57]. One interesting observation, although with unclear consequences, is that mRNA vaccines can induce antibody responses to polyethylene glycol (PEG), likely due to the PEGylated lipids used in the lipid nanoparticle formulations [58, 59]. While it is unlikely that those responses are connected to reactogenicity, they may impact on the efficacy of PEGylated drugs or future mRNA vaccines. Therefore, these responses should be monitored.

## Flexibility of mRNA-based vaccines

For many classical vaccine platforms, the production process needs to be adapted for each antigen. This can be problematic and time-consuming and even small changes in the antigen may lead to several-fold lower yields or unstable products (e.g., [60–64]). As an example, in our laboratory, recombinant spike proteins of Omicron variants are less stable and harder to produce than, e.g., wild-type SARS-CoV-2 spike. Another example is the variable growth capacity of SARS-CoV-2 variants on different types of production cell lines, e.g., wild-type SARS-CoV-2 can be grown on regular Vero.E6 cells for production of inactivated whole-virus vaccines while Omicron subvariants do not replicate well in these cells [65, 66] and may require a different substrate. However, qualifying a new cell line for production, which supports the growth of Omicron variants better, takes a lot of time and effort. While initial antigen design and target identification are also crucial for technologies like viral vectors, DNA vaccines, and RNA vaccines, they do not suffer from these shortcomings since their production process is (except for difficult or very long sequences) antigen-sequence independent [67]. This could be of course a huge benefit when responding to an emerging virus (although classical vaccine platforms were similarly fast this time—see Table 1). Importantly, this is also a huge benefit when dealing with variable viruses that often change since it allows for fast updates of the vaccine composition to mirror circulating strains. With the emergence of the Omicron variant in November 2021, it became clear that an updated vaccine would likely be beneficial. Pfizer/BioNTech and Moderna started to work on updated versions including monovalent and bivalent vaccines. While the update of the actual vaccine could have been done very quickly, initially clinical trials had to be performed and results from these trials did not become available until late spring/early summer of 2022 [68]. They were then shared with regulatory agencies and were seen as favorable. Interestingly, data presented by Pfizer/BioNTech showed that a monovalent Omicron vaccine worked better in inducing neutralizing antibodies against BA.1 than a bivalent vaccine containing both the wild-type spike and the spike of the Omicron subvariant BA.1 [69]. However, the US FDA asked for bivalent vaccines to be moved forward. Importantly, they asked to move a bivalent vaccine with BA.5—then then dominating variant—to market and not one containing BA.1 which had by then been extinct for months [70]. Both Moderna and Pfizer/BioNTech managed to produce these new vaccines within approximately 2 months and they became available in September of 2022 [70]. This demonstrates the speed at which mRNA vaccines can be updated to respond to drifting viruses. It also suggests that a vaccine against a new emerging virus could be manufactured for use in the population within this time frame of 2 months. Of course, it is likely that a vaccine against a new virus would need to undergo all clinical phases again but, e.g., a vaccine against a related sarbecovirus (or even a different betacoronavirus) could be seen as a strain change [71] if regulators are willing to allow this flexibility. This may of course also depend on the pathogenicity of an emerging virus. For a virus with a low case fatality rate, the approach will likely be more conservative while for a virus with a case fatality rate in the range above 10% a faster “strain change” based approach will perhaps be acceptable.

## Pre-fusion spike constructs

Many glycoproteins of enveloped viruses work like a spring-loaded mechanism to trigger fusion between viral membranes and cellular membranes [72]. Typically, the glycoprotein sits in the viral membrane via a transmembrane domain and it also features a fusion peptide which is hydrophobic and is usually hidden in a hydrophobic pocket within the glycoprotein at the pre-fusion stage. A trigger, often the reduction in pH in the endosome after uptake of the virus, then induces a conformation change, which exposes the fusion peptide that then gets inserted into the cellular (endosomal) membrane and the switch to the post-fusion conformation brings the membranes closer and eventually fuses them. This allows the release of genetic material from inside the virion into the cytoplasm and virus replication can begin. Conformational changes from pre- to post-fusion are often very dramatic, in many cases leading to a complete remodeling of the protein involving changes of pre-fusion loop structures to post-fusion helices. Surfaces which may be sites of vulnerability for antibody-based neutralization may completely disappear in the post-fusion conformation, and therefore, the post-fusion conformation may be less than ideal in inducing neutralizing antibodies. Many viral fusion proteins are meta-stable and both the pre- and post-fusion conformation may be present on the virus surface, especially if treated harshly during the vaccine production process [73]. Similarly, recombinant glycoproteins may be partially in the post-fusion conformation. This has been a problem for coronavirus spike proteins—the main target of neutralizing antibodies—as well. The spike protein is typically cleaved between S1 and S2 (and often also at the S2’ site [74]) which “arms” the spring-loaded mechanism [73, 75]. In order to produce homogeneous immunogens in the pre-fusion conformation, the spike protein has to be engineered. Several options have been developed over time for different glycoproteins including disulfide bridges that stabilize the protein, removal of cleavage sites that prevent “arming” of the spring-loaded mechanism, space-filling mutations, salt-bridges, and introduction of prolines—which break helices—into pre-fusion loops that usually become α-helices post-fusion. This last modification had been tested for spike proteins of MERS-CoV, SARS-CoV-1, and HKU1 before the pandemic [15]. Introducing prolines into these spikes let to expression of homogenous pre-fusion spikes [15]. These modifications, the change of lysine and valine in positions 986 and 987 into prolines, were also applied to SARS-CoV-2 vaccine constructs, albeit not all vaccine developers used them or could use them (Table 1). A small number of studies tested the “2P” versions head to head with unmodified versions and those studies found advantages of the modified versus unmodified versions (e.g., [76]). However, this comparison was not performed for all initial vaccine candidates. Importantly, during structural studies, it became clear that while the introduction of the 2P mutations resulted in homogeneous pre-fusion protein for many coronavirus spikes, these two modifications were not enough to result in homogeneous SARS-CoV-2 recombinant spike protein [77]. Additional approaches let to more stable versions of the spike protein including the 6P or “HexaPro” constructs or the “VFLIP” constructs which show high stability and express well [77, 78]. In general, it seems that stabilized pre-fusion constructs for SARS-CoV-2 fare better in terms of immunogenicity than non-stabilized versions [76, 79], and this concept has also been applied to other viral glycoproteins, e.g., the fusion protein of RSV. However, it also needs to be mentioned that SARS-CoV-2 vaccines without these modifications, e.g., the AstraZeneca vaccine or all inactivated vaccine candidates fared very well too in terms of efficacy and effectiveness [44]. A summary of modifications of select vaccine candidates can be found in Table 1.

## Correlates of protection

Correlates of protection can be a very useful tool for development of vaccines, patient management and for understanding resistance to a given virus in the population. They have been established for many vaccines/infections and there are several types including mechanistic correlates of protection and non-mechanistic correlates of protection [80, 81]. Mechanistic correlates of protection describe markers that directly provide protection (e.g., neutralizing antibodies) while non-mechanistic correlates may just correlate with protection but may not cause it. In many cases, several types of immune responses can contribute to protection; they may co-correlate and usually only one of them is established as correlate of protection. Often, this is an antibody titer since antibodies are often directly involved in protection and because they can be easily measured in serum samples. From the beginning of the COVID-19 pandemic, it was assumed that antibodies that target the spike protein and neutralize SARS-CoV-2 in vitro would correlate with protection from symptomatic disease. This was also shown in mechanistic studies in non-human primates [82] and humans [83] but of course other immune responses contribute to protections as well. Efforts to establish neutralizing as well as binding antibody levels as correlate of protection were successful, at least at the academic level [84–87]. However, their implementation was hampered by lack of standardization of immune assays as well as by the occurrence of variants of concern. Immune assays ore often established early and are needed even for early-stage phase I trials. In order to standardize them across the globe, an international standard is needed. While this standard was provided late in 2020 and helped in getting more standardized readouts [88], it arguably came too late and many assays had already been established without it, making comparisons between studies—both clinical trials and academic studies—difficult. However, antibody titers have now been used for licensure of several vaccines based on immuno-bridging (Table 1). Another issue is, that binding antibody against wild-type spike protein (which is readily measured) and neutralizing antibodies (which are harder to measure) correlated well in the beginning of the pandemic [89] but with the emergence of viral variants—especially with the Omicron lineage—neutralization titers dropped off steeply while binding antibody titers were not affected as much [32]. This changed the slope of the curve for the correlation between binding and neutralizing antibodies, making it even harder to interpret results and determine protective titers. In general, it has been hard to implement a correlate of protection for patient management since there is no absolute protective threshold, just a reduction in risk with increasing antibody levels and this is not easily communicated to and understood by physicians and patients. In addition, protection afforded by neutralizing antibodies can also be influenced by the dose of virus a person is exposed too (which in turn is influenced by the immune status of the person who shed the virus, by viral properties and by countermeasures like masks) and likely also other viral factors like the fusogenicity of the spike protein, the replication competence in the upper respiratory tract, the ability to counteract innate, and adaptive immune responses as well as the incubation time (which became shorter over time and therefore leaves less time for an anamnestic response to clear the virus before symptoms arise [27]). These parameters changed over time and are difficult to take into account. Furthermore, protection from infection is likely mostly determined by titers of neutralizing antibodies on the mucosal surfaces of the upper respiratory tract. In summary, possible correlates of protection should be closely monitored and evaluated right away in the beginning of the next pandemic, standards need to become available quicker, and we need to learn much more about the fine details of interactions between the immune system and the virus, especially at mucosal surfaces. Perhaps studying correlates of protection from respiratory viruses in general in more detail would be helpful to respond better to future pandemics.

## Mucosal immunity

In general, the major type of antibody in the lower respiratory tract is IgG which ends up there through transudation or diffusion from serum [90]. A robust IgG response in the lower respiratory tract is hypothesized to be needed for protection from severe disease. Injected vaccines provide this protection efficiently through induction of systemic IgG. However, in order to protect from infection, a neutralizing antibody response in the upper respiratory tract, where the virus will land and establishes infection, is needed. There, IgA, specifically secretory IgA (sIgA) is more abundant [90, 91]. This sIgA is produced by plasma cells in the lamina propria and actively transported onto mucosal surfaces. Mucosal vaccination (nasal, oral, etc.) is required to drive an efficient mucosal immune response in the upper respiratory tract through induction of sIgA [92]. Similarly, it is thought that mucosal vaccination is more efficient to induce local tissue resident memory T cells which can eliminate infected cells quickly [93]. However, it is important to mention that intramuscular vaccination can induce some mucosal IgG antibody responses in the upper respiratory tract too. This IgG is derived from serum and ends up in the upper respiratory tract via transudation and in the case of saliva also through crevicular fluid and microlesions [94]. However, these IgG are just a small fraction of serum IgG antibody titers, and as serum antibody titers wane, these mucosal IgG probably fall below a protective threshold quickly [23].

For COVID-19, the initial prediction was that injected vaccines would not provide protection from infection with SARS-CoV-2 [14]. However, early data from the Moderna clinical trial suggested a high efficacy against infection as well [20]. This protection from infection was likely driven by high titers of serum IgG that made it initially to the upper respiratory tract in sufficient quantities (as described above). However, with waning of the initial antibody peak in serum and with the emergence of variants that were able to escape from neutralizing antibodies to some degree, this protection—as well as protection from symptomatic infection—waned over time (as discussed in the “SARS-CoV-2 vaccine development and implementation” section). However, it has been shown that natural infection with SARS-CoV-2 can induce sIgA responses in the upper respiratory tract [94] and some studies indicate that these responses can be boosted by intramuscular vaccination [23, 95, 96]. In general, it has now been shown by a number of studies that there is an association between (IgA) antibodies in the upper respiratory tract and protection from infection [95–97]. Of course, this type of protection also becomes weaker with variants that escape neutralization. However, sIgA may be less affected by immune escape since it has higher avidity than monomeric IgG [98, 99].

Despite moderate boosting of mucosal immunity by injected vaccines observed in some studies [23, 95], it is likely that local stimulation via mucosal immunization would induce a more robust protection from infection. Development of mucosal SARS-CoV-2 vaccines lagged behind development of injected vaccines considerably and some of these candidates still lack proper funding for further development. Mucosal vaccines have always been the stepchild for vaccinology and there are several reasons for that. When given to naïve individuals, they often induce low serum antibody responses making it hard to establish a correlate of protection. Additionally, mucosal immune responses are more difficult to measure and standardize than serum antibody responses. Finally, especially intranasal vaccines need careful safety evaluation because they are administered close to facial nerves and the brain. While it was flagged early that mucosal vaccines may be needed (also based on pre-clinical experiments in non-human primates [14]), they became on vogue only after widespread breakthrough infections were observed. Now, a number of candidates are in clinical testing (Table 2) and some candidates show efficient protection from infection in animal models (e.g., [100]). Two candidates, both based on adenovirus vectors, have been licensed in China and India after immuno-bridging studies [101]. Immuno-bridging studies compare the immune response of a vaccine candidate to the responses induced by a licensed vaccine, and the assessment is typically done in serum. If the candidate is as good as a licensed vaccine in inducing these immune markers, it may get licensed without a field efficacy trial. However, studies showing that these vaccines indeed induce robust mucosal immunity and protection from infection and transmission are still missing. One candidate, also based on an adenovirus vector, already failed in clinical trials [102]. The large number of candidates now in clinical development and an increased enthusiasm about mucosal vaccines will hopefully result in significant data regarding the safety and efficacy of these different vaccines. The availability of a large number of adjuvants, including those tested in injected vaccines during the pandemic, may also facilitate mucosal vaccine development for vaccines that require adjuvants. We need to understand if and how efficiently mucosal vaccines block infection and transmission, how long lasting the mucosal immune responses are that they elicit and we need to better understand the effects of mucosal antibody and upper respiratory tract cellular immunity including differences between responses in saliva and nasal secretions. This also requires further development and standardization of sampling techniques and immune assays, which is often not trivial. Making these investments now will likely help us to design much better vaccines against seasonal and emerging respiratory viruses that not just prevent morbidity and mortality but also reduce infection and transmission. It is also important to highlight indirect effects that transmission-blocking vaccines may have, especially for immunocompromised parts of the population who cannot built protective immunity by themselves. By reducing virus circulation in the population through strong mucosal immunity, their risk to get infected and experience a bad outcome will be (indirectly) reduced significantly.

**Table 2:** Overview of mucosal COVID-19 vaccines licensed or in clinical development partially based on the WHO COVID-19 vaccine tracker and landscape [161]

Vaccine (company)	Route of administration	Status	Platform and antigen
AD5-nCOV (CanSino)	Inhaled	Licensed in China	Ad5 vectored, spike
BBV154 (Bharat)	Intranasal	Licensed in India	Chimpanzee adenovirus vectored, spike
DelNS1-2019-nCoV-RBD-OPT1 (University of Hong Kong, Beijing Wantai Biological Pharmacy)	Intranasal	Phase III	Influenza virus delNS1 vector, RBD
COVI-VAC (Codagenix/Serum Institute of India)	Intranasal	Phase III	Live attenuated SARS-CoV-2 (attenuated through codon deoptimization)
AVX/COVID-12 (Avimex)	Intranasal	Phase II	Newcastle disease virus, spike
VXA-CoV2-1 (Vaxart)	Oral	Phase II	Ad5 vectored, spike
CIGB-669 (Center for Genetic Engineering and Biotechnology)	Intranasal	Phase I/II	RBD plus hepatitis B virus antigen
*Covishield*, *Vaxzevria*, *AZD1222 (University of Oxford, AstraZeneca)*	*Intranasal*	*Phase I*, *abandoned*	*ChAdOx vector*, *spike*
NDV-HXP-S (CastleVax, Icahn School of Medicine at Mount Sinai	Intranasal	Phase I	Newcastle disease virus, spike
bacTRL-Spike oral DNA vaccine (Symvivo)	Oral	Phase I	*Bifidobacterium longum*-based gene transfer into human cells, spike
*AdCOVID (Altimmune)*	*Intranasal*	*Phase I*, *abandoned*	*Adenovirus vectored*, *RBD*
MV-014–212 (Meissa Vaccines)	Intranasal	Phase I	Attenuated RSV vector, spike
CoV2-OGEN1 (USSF/Vaxform)	Oral	Phase I	Recombinant protein, spike-NP fusion protein
CVXGA1 (CyanVac)	Intranasal	Phase I	Parainfluenza 5 vectored, spike
SARS-COV2 (DreamTec Research)	Oral	Unclear	Bacillus subtilis spores, RBD
Ad5-triCoV/Mac or ChAd-triCoV/Ma (McMasters University)	Aerosolized	Phase I	Adenovirus vectored, spike, NP, and polymerase
MVA-SARS-2-ST (Hannover Medical School)	Inhaled	Phase I	MVA, spike
ACM-001 (ACM Biolabs)	Intranasal	Phase I	Recombinant protein, spike
OMV-linked HexaPro spike vaccine (Intravacc)	Intranasal	Phase I	Outer membrane vesicles (OMVs) from *Neisseria meningitides* linked to recombinant spike

## Universal vaccines against respiratory viruses

**Fig. 1:**
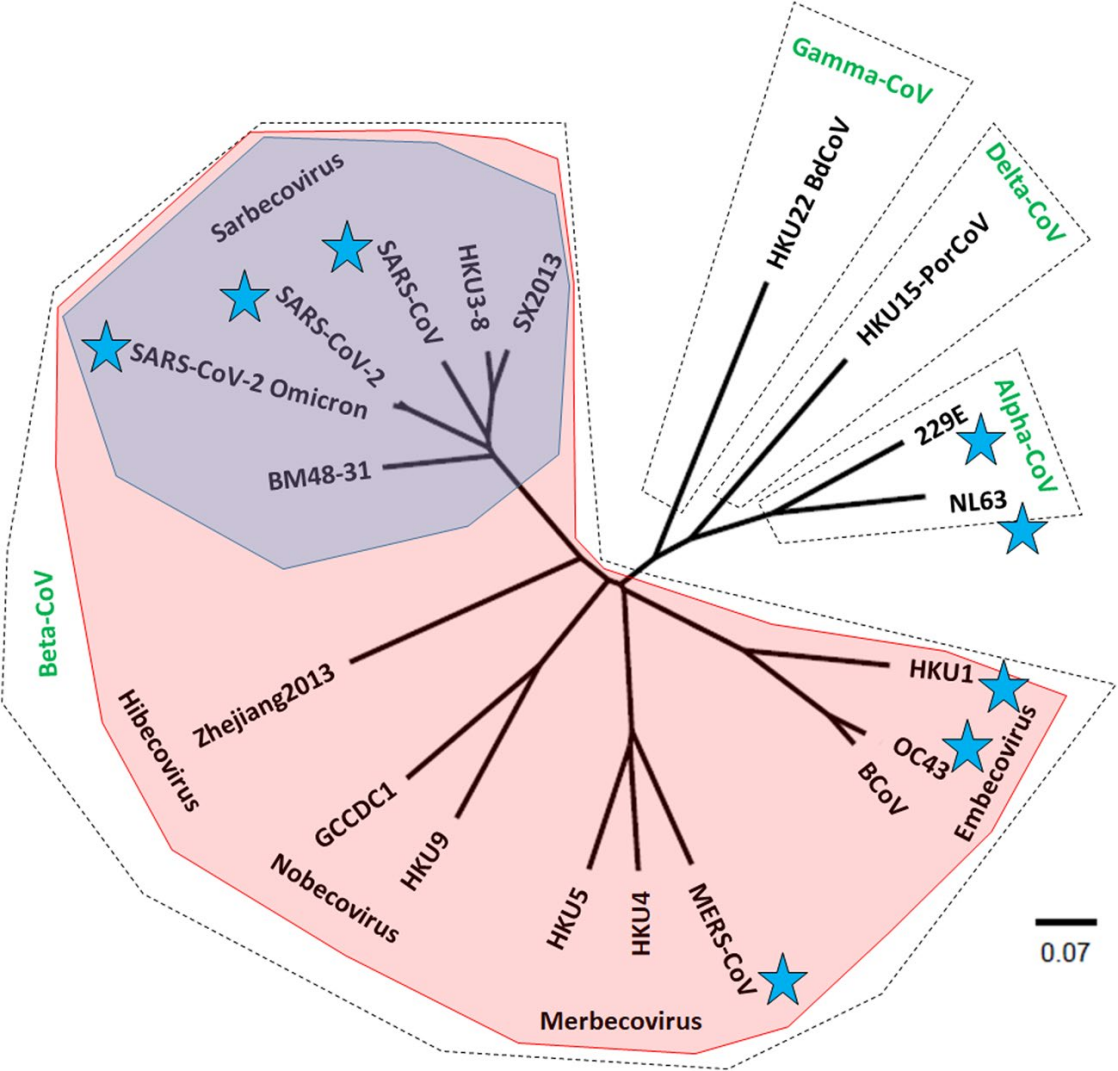
Phylogenetic tree of the *Orthocoronavirinae* based on the amino acid sequence of the spike protein. The tree was built in Clustal Omega, visualized in FigTree and annotated in Microsoft PowerPoint. The scale bare represents a 7% change in amino acids. Alpha-CoV alphacoronaviruses, Beta-CoV betacoronaviruses, Gamma-CoV gammacoronaviruses, Delta-CoV deltacoronaviruses. Viruses marked with blue stars are either circulating in humans or are known to be human pathogenic. BM48-31, bat coronavirus BM48-31/BGR/2008, GenBank# YP_003858584.1; SARS-CoV, Urbani strain, Genbank# AAP13441.1; SARS-CoV-2, ancestral strain, Genbank# BCN86353.1; HKU3-8, Bat SARS coronavirus HKU3-8, Genbank# ADE34766.1; SX2013, BtRf-BetaCoV/SX2013, Genbank# AIA62300.1; BCoV, bovine coronavirus, GenBank# AAA66399.1; MERS-CoV, MERS-CoV camel/Kenya/C1272/2018, Genbank# AXP07355.1; HKU4, *Tylonycteris* bat coronavirus HKU4, Genbank# YP_001039953.1; HKU5, *Pipistrellus* bat coronavirus HKU5, Genbank# YP_001039962.1; HKU9, *Rousettus* bat coronavirus HKU9, YP_001039971.1; GCCDC1, *Rousettus* bat coronavirus GCCDC1, Genbank# QKF94914.1; Zhejiang2013, bat Hp-betacoronavirus/Zhejiang2013, Genbank# YP_009915659.1; HKU15-PorCov, porcine coronavirus HKU15 strain HKU15-155, YP_009513021.1; HKU22-BdCoV, bottlenose dolphin coronavirus HKU22 isolate CF090331, Genbank# AHB63508.1; SARS-CoV-2 Omicron, SARS-CoV-2/human/AUT/SKV-282/2021, Genbank# UFT26501.1; 229E, human coronavirus 229E, Genbank# NP_073551.1; NL63, human coronavirus NL63 isolate NL63/RPTEC/2004, Genbank# AFV53148.1; OC43, human coronavirus OC43 isolate HCoV-OC43/FRA_EPI/Caen/2009/11, Genbank# KF963240.1; HKU1, human coronavirus HKU1 strain HKU1/human/USA/HKU1-12/2010, Genbank# AGW27881.1

The emergence of virus variants in late 2020 [103, 104] and the loss of vaccine effectiveness for symptomatic disease, especially against Omicron, inspired the development of “universal coronavirus vaccines” that would provide broad protection against diverse strains. However, it is important to have clear definitions of what such vaccines should do. First, the coronavirus family is very diverse and contains the *Letovirinae*, *Pitovirinae*, and *Orthocoronavirinae* subfamilies (see https://ictv.global/taxonomy). The *Orthocoronavirinae* subfamily itself contains four very diverse genera (Fig. 1): *Alpha*-, *Beta*-, *Gamma*-, and *Deltacoronavirus*. SARS-CoV-2, SARS-CoV-1, and MERS-CoV are part of the betacoronaviruses. So are the human seasonal coronaviruses OC43 and HKU1. While OC43 and HKU1 are part of the subgenus of embecoviruses, MERS-CoV belongs to the merbecoviruses and SARS-CoV-2 and SARS-CoV-1 belong to the sarbecoviruses. In addition, there are two more subgenera in the betacoronavirus genus: the nobecoviruses and the hibecoviruses. Now, even within the sarbecoviruses, the diversity is relatively high and not all members of the family use ACE2 as receptor [105]. In fact, many of the receptors of different sarbecoviruses are unknown. This means that their receptor-binding domains are relatively divers. It has been shown that infection with SARS-CoV-1 followed by SARS-CoV-2 vaccination can induce antibodies that broadly neutralize ACE2 binding sarbecoviruses and it is therefore likely feasible, to design vaccine regimens that induce similarly broad immunity [106]. However, unfortunately, several mAbs that neutralized SARS-CoV-1 and SARS-CoV-2 where unable to neutralize advanced SARS-CoV-2 variants (e.g., S2X259 [107, 108]). The reason for this is that some epitopes may be seemingly conserved but can change quickly once under immune pressure. It may therefore be a little bit more challenging to design variant-proof SARS-CoV-2 vaccines that protect from all future variants. The next level would be to design vaccines against diverse betacoronaviruses including human seasonal coronaviruses and merbecoviruses, which are a pandemic threat. This is even more challenging since it is unlikely that RBD-targeting immunity would be broad enough. Likely, the focus here would need to be on the more conserved S2 subunit. MAbs against the S2 have been isolated and S2-based cross-reactivity within betacoronaviruses has been reported, so there is at least a molecular basis suggesting that this could work, although it may be difficult [109–112]. Finally, there are now classes of mAbs that recognize the fusion peptide of coronavirus spikes and cross-neutralize alpha- and beta coronaviruses [113, 114]. These antibodies are rare and it is unclear if they can be induced to high enough titers to protect. However, a vaccine which could do this may be a truly universal coronavirus vaccine protecting from all known coronaviruses. Of course, the epitope/antigenic determinants of such a fusion peptide focused universal vaccine will need to be in-depth characterized and selected before vaccine development can begin. One important remaining question is, if it is even necessary to cover all this diversity since it is likely that most of the viruses in the four genera may not have pandemic potential. However, this is just a preliminary assessment based on detected zoonotic infections, much more research into the pandemic potential of these viruses is required.

So far, several promising candidates have been developed. They include three classes: vaccines that contain a variety of RBD or spike proteins from different strains and isolates [115, 116], vaccines based on the S2 of the spike protein [117, 118], and consensus sequence approaches [119]. These approaches are similar to approaches taken for broadly protective or universal influenza virus vaccines and for HIV-1 vaccines [120, 121]. In addition, it may be wise to consider approaches that are less focused on antibody-based protection but provide T-cell based cross-protection. Such vaccines may be infection permissive but may provide substantial protection from sever disease. Suitable targets for these approaches remain to be identified but, e.g., more conserved internal proteins like the nucleoprotein have been proposed as T-cell vaccine targets [122]. What is important to realize here is that these approaches will not lead to easy and quick success as initial SARS-CoV-2 vaccine development did. One reason for this is the absence of Operation Warp Speed-like funding and lack of a pandemic situation, which required prioritization of vaccine development on all levels. Second, the immunological problems that need to be solved with universal vaccines are very different in nature from the simple induction of antibodies and T-cells to a single wild-type spike. Nevertheless, we need to make an effort to try to develop these vaccines, even if it takes significant time and resources, since they would be excellent tools for pandemic preparedness and for protecting the population from ever-evolving variants.

## Global access

**Fig. 2:**
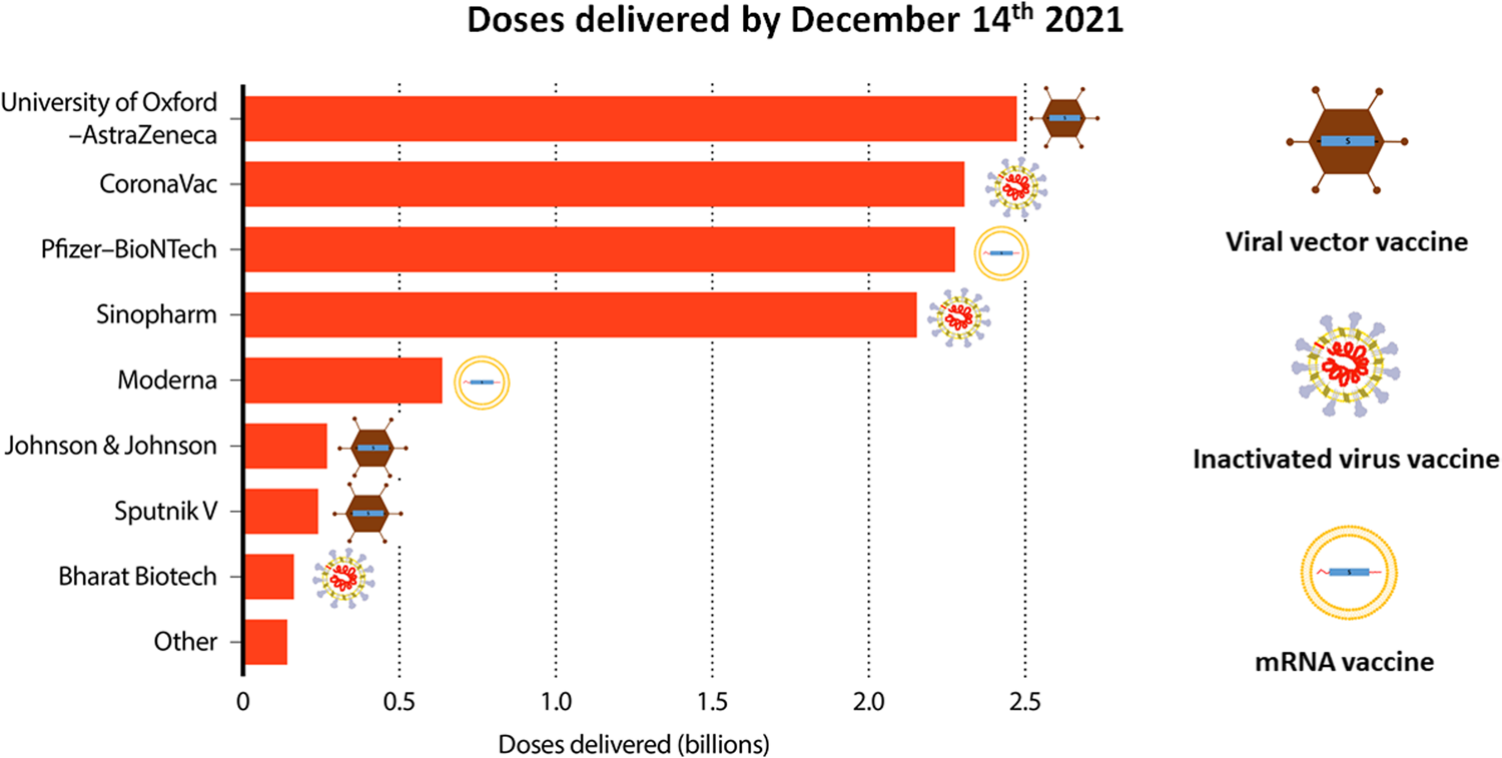
Vaccine doses used by December 2021 by producer and technology. The figure is adapted from an original figure published in Nature and is based on data from Airfinity

As soon as the causative agent of COVID-19 was identified, a race to develop vaccines began across the globe and, as described above, many different vaccine technologies were employed. mRNA vaccines were the first to be rolled out at large scale with vectored vaccines following suit quickly. Inactivated vaccines were also not far behind. Since mRNA vaccines were developed in Europe and North America with governments there making large investments upfront, a lot of the mRNA vaccine produced initially ended up in Europe and North America as well. Distribution of vectored and inactivated vaccines was much more equal across the globe. In the end of 2021, in the phase where vaccines and timely vaccine rollout really made a big difference, most of the vaccine doses used were actually either inactivated or vectored vaccines (Fig. 2). While in Europe and North America mRNA vaccines are seen as the platform that made the main impact, it needs to be kept in mind that these vaccines were initially—to say it in a provocative way—“boutique vaccines” for rich customers. But it was other types of vaccines which likely saved more lives across the globe.

Equal vaccine distribution was and is of course a problem in more general terms also. Early in the pandemic, it was anticipated that wealthy countries would try to buy available vaccine doses for their own populations first. To counteract this, the Coalition for Epidemic Preparedness Innovations (CEPI), the Global Alliance for Vaccines and Immunization (GAVI), and the World Health Organization (WHO) initiated COVID-19 Vaccines Global Access (COVAX) [123] which was charged with making sure that vaccines were distributed more equally across the globe. While COVAX had some effect, the anticipated scenario that wealthy nations would buy up the vaccine supply became more or less a reality. Low- and middle-income countries (LMICs) often had to wait for much longer and many times were supplied with vectored and inactivated vaccines produced in China or India, which had somewhat lower efficacy (but still had very high efficacy against severe disease as discussed above). Delayed vaccine delivery often also had a negative impact on vaccine hesitancy. For example, several European countries detected thrombotic events following vaccination with vectored vaccines early during rollout due to excellent surveillance systems [55]. These severe but rare events where highly publicized and in some countries triggered a switch in favor of mRNA vaccines. As a consequence, LMICS perhaps had more supply of vectored vaccines and less of mRNA vaccines but the bad reputation of vectored vaccines at this point—based on data from high-income countries—may have negatively impacted vaccine uptake in low-income countries.

In summary, unequal distribution and staggered rollout of vaccines across the globe caused severe issues and had likely a negative impact on vaccination rates but also vaccine uptake in LMICs. Several factors contribute to these problems: First, centralized production of vaccines in only a few, often high income, countries favors “vaccine politics” and “vaccine grapping.” This could be solved by establishing local vaccine production facilities that can produce vaccine regionally without dependence on entities in high-income countries or control of vaccine distribution by single countries or companies. It is also likely, that locally produced and trialed vaccines have initially more trust by the local population since they are seen as their “own” vaccine. Several countries seem to implement this strategy including Brazil, Thailand, Vietnam, and Indonesia. The second factor is of course local innovation and know how. A common theme discussed during the pandemic is that intellectual property rights curtailed countries from making, e.g., their own mRNA vaccines. However, during an emergency, countries can elect to compulsory license IP rights, according to the Agreement on Trade-Related Aspects of Intellectual Property Rights (TRIPS) of the World Trade Organization. To the author’s knowledge, this has not been applied during the COVID-19 pandemic for vaccines. There may be several reasons for this but one is missing know how. Even of IP rights are waived for a technology, it does not mean that a certain vaccine can be produced easily. Production processes for vaccines are complicated, reagents need to be available in a certain quality (good manufacturing practice (GMP) quality), and small changes in any process parameters may have a huge impact and could necessitate further clinical testing. However, just because IP rights are waived, it does not mean that access to know how is available or that the “generic” product is similar enough to rely on safety and efficacy data from the original product. It would therefore be important to build local centers of innovation in vaccinology as well as clinical trial centers which can generate this know how and implement it quickly when a new pandemic virus emerges. Of course, it would also be helpful if vaccine developers in high-income countries would share their know how, but commercial realities will likely make this a rare case. It is heartening to see that several countries are taking steps now to advance their regional vaccine innovation and development capacities to be better prepared and independent during the next pandemic.

## The elephant in the room: vaccine hesitancy and anti-vaxxers

Medications are usually sought to treat symptomatic disease and to get better and many patients do not worry too much about side effects that medicines can have because they are usually less problematic than the disease symptoms that they experience. This is different for vaccines. Vaccines are taken as a prophylactic to prevent disease (or severe disease) that may occur in the future. However, usually, they are used in currently healthy individuals. People are therefore much more wary about side effects that those vaccines can have, even if the side effects are rare. This, in addition to insufficient or wrong information and anecdotes about vaccine injuries, can lead to an unbalanced assessment of risk of vaccination versus risk of infection. In addition, many people are worried about new technologies that have not been used at large scale and for a long time in humans. Many assumed that mRNA vaccines were tested for the first time in humans for COVID-19 which is of course not correct since several trials had already been conducted before 2020 [124]. In addition, there is a general misunderstanding about when severe side effects of vaccines are expected. A major theme in the population is the worry that vaccines may cause issues many years after they have been given. This is of course very unlikely. Major (and rare) side effects of vaccines are typically materializing within weeks or months post-vaccination and are either caused by the vaccine directly (e.g., an attenuated virus reverting or being virulent in an immunocompromised individuals—oral polio vaccine, yellow fever vaccine, etc.) or by an overreacting immune system (e.g., Guillain–Barre syndrome with influenza vaccines, narcolepsy with pandemic H1N1 vaccine). In both cases, symptoms arise mostly within a short period of time after vaccination [125–127]. Issues that arise many years post-vaccination have not been reported and it would be hard to think of mechanisms for those. Another important point that is hard to communicate is the inability to detect rare severe side effects in phase III clinical trials. Severe thrombotic events, as detected for adenovirus vector-based SARS-CoV-2 vaccines, and myocarditis as detected for mRNA vaccines and anaphylactic reactions as detected for both, are too rare to be detected in a phase III trial and can only be detected in phase IV trials or using post-marketing surveillance systems. When these effects then showed up and were reported in 2021, the population was concerned. Communication about this ahead of time, including how this would be handled and that this is something seen for many vaccines, would perhaps have helped to reduce vaccine hesitancy. Especially during a pandemic, when a lot of misinformation is circulating and when there is a lot of anxiety in the population, proper communication ahead of time is important to make sure people are well informed and can make the right decisions. That is certainly an important lesson learned from the COVID-19 pandemic where this was not optimally implemented in many countries, although positive examples like, e.g., the COVID-19 communication in New Zealand, exist [128]. Another lesson here is that we, as vaccinologists, virologists and immunologists are amateurs when it comes to understanding vaccine hesitancy and optimal communication. We need to foster collaborations with psychologists, sociologists, and communication experts to really understand what drives people’s anxiety and how information is properly communicated. Helpful literature in that respect has been published (e.g., [129, 130]).

A somewhat different issue is the issue of anti-vaxxers. In contrast to people who are hesitant to get vaccinated, anti-vaxxer believe vaccines are detrimental in general. The anti-vaccine movement is not a recent phenomenon, it is as old as vaccines themselves [131]. However, themes used by anti-vaxxers have changed over time. In addition, it seems that the movement gained significant traction during the COVID-19 pandemic, fueled by misinformation and anxiety. The hypothesis often is that vaccines damage the body but are pushed by governments and pharmaceutical companies to make money. This sometimes includes conspiracy theories hypothesizing that causative agents of disease were released on purpose so that vaccines can be presented as solution—again to make money. Other common themes that often come up are that vaccines cause infertility or different types of severe diseases, that they contain a chip so that the government, or some dark hidden entities can control us etc. Especially rumors about negative effects on fertility generate fear across the population and maybe inclusion of pregnant women in phase III trials could dampen these worries during the next pandemic. It is not always clear, what the real motivation of anti-vaxxers is, if they actually believe the misinformation they spread or if there are hidden economic motifs involved as well. Of course, information spread by anti-vaxxers feeds the fears of vaccine hesitant individuals and can have a huge impact on efforts to roll out vaccines. In some cases, the anti-vax movement also used violence against scientists and medical personnel. An important example is the case of an Austrian physician who advocated for COVID-19 vaccines. She received significant death threats against her and her personnel and was forced to close her practice due to security concerns and, left without help from the authorities, committed suicide [132].

It seems that the anti-vax movement as well as vaccine hesitancy grew significantly during the COVID-19 pandemic, despite the success of COVID-19 vaccines and their excellent safety profiles. Unfortunately, this may have a negative impact on routine vaccination which in turn may lead to an increase in vaccine preventable diseases in the population as evidenced, e.g., by a polio case in New York in 2022 [133]. Furthermore, it is very likely that vaccine uptake during the next pandemic will be even lower than during this pandemic. A combined effort by vaccinologists, immunologists, virologists, psychologists, sociologists, communication experts, policy-makers, politicians, and authorities is necessary to counteract this phenomenon. In this context, it would be advisable to provide professional training to scientist and health care workers on how to address the anti-vax movement in public. Of note, both vaccine hesitancy and anti-vaxxers are a problem in high-income and low-income countries although root causes may be different depending on the country.

## Summary and outlook

We have learned a lot during the SARS-CoV-2 pandemic. Countermeasures were developed in record time, vaccine technologies were tested side-by-side, and novel technologies were rolled out at large scale for the first time. From a technology point of view, we responded quickly—even though a quicker response is, and would have been possible [71]. Looking forward into the future, we could be well prepared for new pandemics. Sequencing technologies and high-throughout serology allow for effective surveillance in animal and human populations. Virology laboratories are set up well to connect genotypes with concerning pathogen phenotypes to allow for establishment of early warning systems. We have all the tools necessary to understand correlates of protection if needed and vaccine technologies for quick employment in the case of a new pandemic have been developed and are available. In summary, we understand the key issues that need to be addressed for pandemic preparedness from a scientific and technological point of view. Nevertheless, the outlook into the future is a negative one. Just about 3 years after the emergence of SARS-CoV-2, it seems politicians and governments have forgotten that the pandemic existed and very little is spent—e.g., in comparison to defense—on pandemic preparedness. This is despite the fact that enormous amounts of money were lost during the pandemic [134] and despite the fact that spending on pandemic preparedness could certainly be seen as defense spending. In addition, beliefs in conspiracy theories, denial, and anti-vax sentiments have spread far and wide, and may make it difficult to get “buy in” from the population once the next pandemic occurs. Unfortunately, this will likely be sooner than later. Several factors including a larger human animal interface (due to a growing population and therefore a growing number of domestic animals), ecosystem destruction, climate change, and others will likely lead to a higher frequency of outbreaks compared to the past. We have learned a lot from the SARS-CoV-2 pandemic. As a society, we now need to start to take viruses seriously and implement what we learned.
